# Amount and Frequency of Added Sugars Intake and Their Associations with Dental Caries in United States Adults

**DOI:** 10.3390/ijerph19084511

**Published:** 2022-04-08

**Authors:** Norah Alosaimi, Eduardo Bernabé

**Affiliations:** Dental Public Health Group, Faculty of Dentistry, Oral & Craniofacial Sciences, King’s College London, Bessemer Road, London SE5 9RS, UK; norah.alosaimi@kcl.ac.uk

**Keywords:** dental caries, dietary sugars, nutrition assessment, cross-sectional studies, adult, United States

## Abstract

The relative importance of amount and frequency of sugars intake for caries development has been a matter of debate in recent years, yet only one study has formally evaluated this question among adults. The aims of this study were to explore the shape of the relationship between amount and frequency of added sugars intake and their associations with dental caries among adults. Cross-sectional data from 10,514 adults, aged 20+ years, from the National Health and Nutrition Examination Survey (NHANES) 2011–2016 were analyzed. The amount (g/day) and frequency (items/day and episodes/day) of added sugars intake were derived from dietary recalls. Dental caries was indicated by the DMFS and DS scores. Fractional polynomials were used to characterize the relationship between amount and frequency of added sugars intake. Their associations with DMFS and DS were evaluated in negative binomial regression models adjusting for confounders. There was a logarithmic relationship between amount and frequency of added sugars intake. The amount of added sugars intake was positively associated with the DMFS (rate ratio: 1.11, 95% CI: 1.07–1.15) and DS scores (1.43, 95% CI: 1.33–1.54). However, the estimates for frequency of added sugars intake varied depending on how it was expressed. When expressed in items/day, it was not associated with the DMFS (1.02, 95% CI: 0.99–1.04) or DS score (0.91, 95% CI: 0.81–1.02). When expressed in episodes/day, it was positively associated with the DMFS (1.43, 95% CI: 1.33–1.54) but not with the DS score (0.95, 95% CI: 0.86–1.04). This study found a curvilinear relationship between the amount and frequency of added sugars intake. Furthermore, the amount of added sugars intake was more consistently and strongly associated with dental caries than the frequency of intake.

## 1. Introduction

Dental caries is a biofilm-mediated, sugar-driven, multifactorial, dynamic disease that results in the phasic demineralization and remineralization of dental hard tissues [1]. The World Health Organization (WHO) has called on countries to reduce their intake of free sugars below 10% of total energy intake (TEI), with further health benefits when intake is reduced below 5% TEI [2]. These recommendations were informed by a systematic review on the effect on caries of restricted sugar intake [3,4]. Free sugars include monosaccharides and disaccharides added to foods by the manufacturer, cook, or consumer, plus sugars naturally present in honey, syrups, and fruit juices [2]. In the United States, the Dietary Guidance for Americans 2015–2020 recommends that the intake of added sugars should not exceed 10% of TEI [5]. Added sugars include syrups and other caloric sweeteners that are added to foods during preparation, processing, or at the table [5]. The definitions of free and added sugars differ mainly in their respective inclusion or exclusion of sugars in juiced or pureed fruit and vegetables [6]. Both recommendations are based on amount of intake (relative to caloric intake). However, frequency of intake is what dental care professionals are familiar with in their daily practice. Frequency of intake can be measured in different ways, such as counting the number of items containing sugars, eating occasions, or time of the day [7,8,9]. In dentistry, it is common to count eating occasions because that reflects more closely the drop in salivary pH and subsequent demineralization of tooth surfaces that occurs after the ingestion of sugars [10,11]. As per the Stephan curve [12], it takes saliva around 20 min to buffer any acids produced from the bacterial fermentation of sugars and return pH to normal levels.

The relative importance of amount and frequency of sugars intake for caries development has been a matter of debate in recent years [10,11,13]. However, only one study has formally evaluated this question among adults by analyzing both amount and frequency simultaneously. An 11-year prospective study in Finland showed that only the amount of total sugars intake remained associated with caries increment when both indicators were simultaneously included as predictors in the regression model. Interestingly, the authors measured frequency of sugars intake as the number of food/beverages containing sugars eaten per day [13]. The association between different indicators of sugars intake is logically high. Thus, determining the relevance of amount versus frequency is challenging because an increase in one component frequently results in a rise in the other [10,11]. To begin with, it would be informative to know what type of relationship exists between these two indicators of sugars intake. Assuming a linear relationship, a correlation of 0.64 between both indicators was reported in adults [13], suggesting that a limited portion of the variance in one indicator (41%) can be explained by the other.

Beyond the shape of their relationship, it is important to know which indicator of sugars intake might be more relevant for caries development as the answer to this question will influence health education, dietary counselling, and preventive strategies on curbing sugars intake to control dental caries. To address these gaps in knowledge, this study explored the relationship between amount and frequency of added sugars intake and their independent associations with dental caries in adults.

## 2. Methods

### 2.1. Data Source

This study analyzed cross-sectional data from the National Health and Nutrition Examination Survey (NHANES) 2011–2016, a series of annual population-based surveys conducted by the Center for Disease Control and Prevention (CDC). NHANES uses stratified multistage probability sampling to recruit a nationally representative sample of the general non-institutionalized population in the United States (US). Certain population subgroups are oversampled to increase the precision of estimates, namely Hispanics, Blacks, and Asians; low-income individuals (<130% of the federal poverty level) and older adults (80+ years). The NHANES cycles from 2011–2016 are the latest cycles to include a comprehensive caries examination. Overall, 9756, 10,175, and 9971 persons were interviewed (response rate: 72.6%, 71.0%, and 61.3%) in 2011–2012, 2013–2014, and 2015–2016, respectively. Of them, 8472, 8966, and 8859 participants, aged one year or above, were clinically examined for oral conditions in each NHANES cycle [14].

A total of 17,048 adults, aged 20 years and above, participated in NHANES 2011–2016. Of them, 4057 and 2801 did not participate in the dietary assessment and dental examination, respectively. Of the remaining 11,621 participants, 1107 were excluded for missing data on poverty income ratio (*n* = 900), dental attendance (*n* = 225), and education (*n* = 6). The final study sample included 10,514 adults.

### 2.2. Measures

Intake of added sugars was determined using the added sugars values in the US Department of Agriculture’s Food Patterns Equivalent Database (USDA FPED) for each NHANES cycle [15]. The FPED defines added sugars as sugars, syrups, or caloric sweeteners that are added to foods as an ingredient during preparation, processing, or at the table. Additionally, fruit juice concentrates used in foods without further dilution are also considered as added sugars in the FPED [16]. Data on all foods and drinks ingested, including their corresponding USDA Food and Nutrient Database for Dietary Studies (FNDDS) code, time and place of eating, quantity (g), and calories, were obtained from the two 24 h dietary recalls in each NHANES cycle (the first carried out in person and the second over the phone 3 to 10 days later) [17]. Three indicators of added sugars intake were created from each recall day, which were then averaged across the two days [18]. One indicator reflected the amount of added sugars intake (g/day). The other two reflected the frequency of added sugars intake expressed in different units, namely the count of all food items containing added sugars eaten in a day (items/day) and the count of eating occasions that contained food items with added sugars but were separated from each other, by at least 20 min, to reflect separate acid attacks as per the Stephan curve (episodes/day).

Clinical oral examinations were performed by trained examiners at the mobile examination center (MEC), which were equipped with a portable dental chair, artificial light, and compressed air. Caries was diagnosed using the Radike criteria. Third molars were not included in the examination. Dental examiners were trained and calibrated prior to data collection. Blinded repeated examinations were carried out during the survey to establish inter-rater reliability. Kappa values for caries experience were 0.93 and 0.96, respectively, and 0.82 and 0.91 for untreated caries [14]. The number of decayed, missing, and filled tooth surfaces (DMFS) and the number of decayed tooth surfaces (DS) were estimated to represent the levels of caries experience (past and present) and untreated disease, respectively.

Several variables were also included in the analysis as they could confound the association between added sugars intake and dental caries (i.e., they were well-established common causes of both exposure and outcome). These variables were demographic factors (sex, age, and race/ethnicity), socioeconomic position (education and poverty income ratio), dental attendance pattern, and TEI (kcal/day). TEI was estimated as the sum of the energy content of all foods and beverages consumed [17]. Dental attendance pattern was determined based on time since last dental visit and the main reason for last dental visit. Participants who visited the dentist for a check-up in the past year were considered regular attenders. Otherwise, they were considered irregular attenders.

### 2.3. Statistical Analysis

All analyses were conducted in Stata IC 16 (StataCorp LP, College Station, TX, USA) incorporating sampling weights and survey features. We first compared the characteristics of our study sample with those of adults excluded due to missing values to assess the impact of missing data. We then explored the shape of the relationship between amount and frequency of added sugars intake using fractional polynomials (FP) as they allow modelling non-linear associations [19,20,21]. FPs differ from regular polynomials in that they allow logarithms, non-integer powers and powers to be repeated [20]. The best fitting first-order polynomial (FP1) was selected among 8 possible powers (−2, −1, −0.5, 0, 0.5, 1, 2, and 3) and the best fitting second-order polynomial (FP2) was selected among 36 possible pairs of the same set of powers. The best FP1 and FP2 models were those with the smallest deviance. Thereafter, the best-fitting FP was chosen through an iterative process of three sequential comparisons: (i) the linear model versus an empty model, (ii) the FP1 model versus the linear model, and (iii) the FP2 model versus the FP1 model [22,23]. If a comparison was significant, the more complex model was preferred. These comparisons were made with the likelihood ratio test based on the deviance differences between models [22,23].

The associations of the amount and frequency of added sugars intake with the DMFS and DS scores were examined in negative binomial regression models as the two caries measures were counts with overdispersion. Rate ratios (RR) with 95% confidence intervals (CI) were reported as the measure of association. All indicators of added sugars intake were standardized (mean = 0, SD = 1) to allow comparison of their coefficients. Two models were presented for each indicator. Model 1 was adjusted for demographic factors (sex, categorical age, and race/ethnicity), socioeconomic measures (education and poverty income ratio), dental attendance pattern, and TEI. The residual method was used to adjust for energy intake [24]. Models 2A and 2B were additionally adjusted for the other indicator of added sugars intake. The frequency of added sugars intake was expressed in items/day in Model 2A and in episodes/day in Model 2B.

## 3. Results

The characteristics of the study sample are shown in Table 1. Differences between the study sample and participants excluded due to missing data were observed. Non-white participants, those from lower socioeconomic backgrounds, and those with lower intake of added sugars were underrepresented in the study sample. The mean DMFS and DS scores were 34.2 (SD: 30.1, range: 0 to 128) and 2.4 (SD: 7.5, range: 0 to 125) surfaces, respectively. The mean amount of added sugars intake was 68.8 g/day (SD: 54.5, range: 0 to 613.1) whereas the mean frequency of added sugars intake was 5.4 (SD: 2.3, range: 0 to 17.5) and 4.1 (SD: 1.1, range: 0 to 9) when expressed in items/day and episodes/day, respectively.

**Figure 1:**
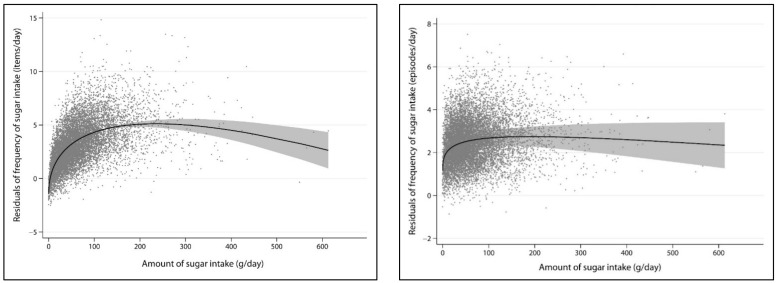
Graphical representation of the best-fitting fractional polynomials to characterize the relationship between the amount and frequency of added sugars intake. Predicted curves with 95% confidence intervals are reported along with the residuals. The frequency of added sugars intake in items/day is shown at the top (powers 0.5; 1) and in episodes/day at the bottom (powers 0.5; 0.5).

Assuming a linear relationship, the amount of added sugars intake was moderately correlated with the frequency of intake expressed in items/day (r = 0.51), but it was weakly correlated with the frequency of intake expressed in episodes/day (r = 0.23). A correlation of 0.56 was found between the two forms of reporting the frequency of added sugars intake. Table 2 describes the results from fitting FP models to the data to characterize the relationship between amount and frequency of added sugars intake while adjusting for confounders. The FP2 with powers (0.5; 1) and (0.5; 0.5) provided the best fit to the data, and therefore, they were preferred to represent the logarithmic relationship observed between amount and frequency of added sugars intake expressed in items/day and episodes/day, respectively (Figure 1). In sensitivity analysis, excluding the participants with intakes of added sugars over 200 g/day (*n* = 312, 3%) did not alter the results. The same set of FP2, with powers (0.5; 1) and (0.5; 0.5), respectively, provided the best fit to the data. Moreover, the correlations of amount of intake with frequency expressed in items/day and episodes/day were slightly weaker (0.43 and 0.19, respectively).

Both the amount and frequency of added sugars intake were associated with the DMFS score after adjustment for confounders (Table 3). In the mutually adjusted model (Model 2A), a 1-SD increase in the amount of added sugars intake (=54.5 g/day) was associated with 1.11 (95% CI: 1.08–1.15) times greater DMFS score whereas a 1-SD increase in the frequency of added sugars intake (=2.3 items/day) was not associated with the DMFS score. In Model 2B, a 1-SD increase in the amount of added sugars intake was associated with 1.11 (95% CI: 1.07–1.15) times greater DMFS score whereas a 1-SD increase in the frequency of added sugars intake (=1.1 episodes/day) was associated with 1.05 (95% CI: 1.02–1.08) times greater DMFS score. Furthermore, the amount but not the frequency of added sugars intake was associated with the DS score after adjustment for confounders. In Model 2A, a 1-SD increase in the amount of added sugars intake was associated with 1.49 (95% CI: 1.38–1.61) times greater DS score whereas a 1-SD increase in the frequency of added sugars intake (items/day) was not associated with the DS score. In Model 2B, a 1-SD increase in the amount of added sugars intake was associated with 1.43 (95% CI: 1.33–1.54) times greater DS score whereas a 1-SD increase in the frequency of added sugars intake (episodes/day) was not associated with the DS score.

## 4. Discussion

This study found a curvilinear relationship between the amount and frequency of added sugars intake. In addition, the amount of added sugars intake was more consistently and strongly associated with dental caries than the frequency of intake.

The first finding of this study was that a curvilinear relationship was the best way to characterize the association between the amount and frequency of added sugars intake. Consistent with a logarithmic curve, there was a steep increase in the frequency of intake in adults consuming less than 50 g/day and much smaller increases in frequency of intake in adults consuming 50 to 100 g/day. The curve reaches a plateau at 100 g/day. Two points are worth noticing: first, many participants exceeded their daily allowance of added sugars (~50 g/day) in few eating occasions, and second, there was great variation in amount of intake among adults with similar frequency of intake. This finding challenges the widely-held assumption that both indicators of sugars intake are highly correlated [11]. The two indicators were weaklier correlated in our study than in a previous study in Finnish adults [13]. Although this difference could be due to methodological differences between studies (total sugars from food frequency questionnaires in the previous study and added sugars from food recalls in the present study), it is possible that contextual factors also play a role as the pattern of sugar consumption varies across world regions [25]. We also found a moderate correlation between two alternative methods to report the frequency of added sugars intake (items/day and episodes/day), suggesting that a considerable amount of information is lost when only considering eating occasions that are at least 20 min apart.

The second finding of this study was that the amount of added sugars intake was more consistently and strongly associated with dental caries than the frequency of added sugars intake. This finding corroborates those from a previous study in Finland [13]. To determine the relative importance of each indicator of added sugars intake in caries etiology, an important requirement is that studies measure both variables simultaneously. Few epidemiological studies have followed this approach. In our study, the amount of added sugars intake was positively associated with greater caries experience and more untreated disease. On the contrary, the estimates for frequency of added sugars intake varied depending on how it was expressed (either as items/day or episodes/day). When reported as items/day, the frequency of added sugars intake was not associated with caries experience or untreated caries. When reported as episodes/day, it was positively associated with caries experience but not with untreated caries. An explanation for this finding is that the ‘true’ intake of added sugars, which is what studies aim to measure, is better reflected by the amount than the frequency of intake.

Whilst population-level targets are typically based on nutrient goals, such as reducing the amount of added sugars intake to <10% TEI [5], some have argued that patient-level targets could be based on reducing the frequency of added sugars intake as this is easier to communicate to patients [26]. We believe that patient-level targets should also be based on curbing the amount of intake because, as shown in this study, the frequency of consumption does not correlate linearly with the amount of consumption. Furthermore, reducing the frequency of added sugars intake alone will not reduce the risk of non-communicable diseases related to excess sugars. Some patients exceed their maximum daily allowance of sugars despite having a low frequency of intake [27]. Liaising with dietitians and nutritionists is crucial to provide appropriate dietary advice at the individual level. Dentists must provide dietary advice that is aligned to that given by other health professionals, either when working with populations or patients. This will facilitate multi-disciplinary work and avoid conflicting messages. Perhaps a way forward is to target the main sources of added sugars in a patient’s diet, such as sugar-sweetened beverages, table sugar, or sweet snacks, following an individualized dietary assessment.

Some recommendations for further research can also be made. First is the role of fluoride exposure. Although the Finnish study showed that daily brushing with fluoride toothpaste moderated the association between amount of sugars intake and DMFT but not that between frequency of sugars intake and DMFT [13], the effect of access to fluoridated water on the relative contribution of amount and frequency of sugars intake requires further testing. Whether the same relationship between amount and frequency of added sugars intake is also found in children and adolescents remains unknown. Furthermore, all evidence on the correlation between amount and frequency to date has only been cross-sectional. Therefore, our findings await confirmation from longitudinal studies including multiple dietary assessments and caries examinations over time. There is a growing interest in the cariogenic role of starches, especially rapidly digested (processed) starches. Recent systematic reviews concluded that robust longitudinal studies on the relationship between starches and dental caries are needed to inform policy [28,29].

This study is not without limitations. First, we analyzed cross-sectional data which precludes us from making any causal inferences on the associations tested. Second, some participants were excluded from the analysis because of missing data. Although we used sampling weights to correct for non-response, the results should be extrapolated with caution due to discrepancies between adults included and excluded from the analysis. In addition, as data were from the US the results might not be generalizable to other countries. Third, our dietary assessment was based on two separate 24 h food recalls. Although dietary recalls provide a comprehensive assessment of the diet of individuals, the data collected may not reflect long-term eating patterns. Finally, we could not evaluate the duration of consumption of items containing added sugars. Although most foods are consumed during discrete time periods, certain items such as sugar-sweetened beverages and highly processed snacks (high in added sugars) are typically consumed throughout the day. This limitation underestimates the frequency of intake and could explain its secondary role, relative to amount of intake, in the present study.

## 5. Conclusions

This study found evidence of a curvilinear relationship between the amount and frequency of added sugars intake. Furthermore, the amount of added sugars intake was more consistently and strongly associated with dental caries than the frequency of intake, after accounting for established risk factors.

## Figures and Tables

**Table 1:** Characteristics of the study sample and comparison against participants excluded because of missing data.

Categorical Variables	Excluded (*n* = 1107)		Study Sample (*n* = 10,514)		*p*-Value ^a^
	*n*	%	*n*	%	
Sex					0.823
Men	563	49.1	5011	48.6	
Women	544	50.9	5503	51.4	
Race/ethnicity					<0.001
White	255	45.2	4358	67.5	
Black	298	17.8	2300	10.5	
Hispanic	374	24.8	2390	14.0	
Mixed/Other	180	12.2	1466	8.1	
Education					<0.001
Less than high school	330	24.0	1848	11.8	
High school	242	22.2	2263	20.3	
More than high school	529	53.9	6403	67.8	
Poverty income ratio					<0.001
0–100%	89	37.3	2185	14.4	
101–200%	69	34.5	2643	20.3	
201–300%	24	12.3	1583	14.9	
301–400%	13	9.0	1207	12.6	
>400%	12	7.0	2896	37.8	
Dental attendance pattern					0.708
Regular	335	45.0	4211	46.2	
Irregular	547	55.0	6303	53.8	
**Numerical Variables**	**Mean**	**(SD)**	**Mean**	**(SD)**	***p*-Value ^a^**
Age, years	46.5	(20.8)	46.7	(16.4)	0.796
Energy intake, kcal/day	1950.4	(911.5)	2107.7	(772.7)	<0.001
Amount of intake ^b^, g/day	61.9	(57.1)	68.8	(54.5)	0.026
Frequency of intake ^b^, items/day	4.8	(2.4)	5.4	(2.3)	<0.001
Frequency of intake ^b^, episodes/day	3.9	(1.3)	4.1	(1.1)	0.003
DMFS, surfaces	31.5	(36.5)	34.2	(30.1)	0.116
DS, surfaces	2.7	(8.5)	2.4	(7.5)	0.340

^a^ Chi-squared was used to compare categorical variables and *t*-test was used to compare continuous variables. ^b^ These indicators refer to intake of added sugars. For reference purposes only, the mean (SD) amount of intake was 108.0 (60.9) g/day whereas the mean frequency of intake was 12.2 (4.2) times/day and 4.5 (1.3) episodes/day, respectively, when total sugars (as opposed to added sugars) were considered.

**Table 2:** Comparison of 44 fractional polynomial (FP) models for the regression of frequency of added sugars intake on amount of added sugars intake and covariates.

Outcome	Models Compared ^a^	df	Deviance	Deviance Difference	*p*-Value	Powers
Frequency of added	Omitted	2	44,092.7	3474.5	<0.001	
sugars intake	Linear	2	42,297.1	1678.9	<0.001	1
items/day	FP1	1	40,774.2	156.0	<0.001	0
	FP2	0	40,618.2	0.0		0.5; 1
Frequency of added	Omitted	2	30,610.3	387.9	<0.001	
sugars intake	Linear	2	30,461.6	239.2	<0.001	1
episodes/day	FP1	1	30,247.4	25.0	<0.001	0
	FP2	0	30,222.5	0.0		0.5; 0.5

FP1: First-degree fractional polynomials; FP2: Second-degree fractional polynomials; df: degrees of freedom. ^a^ All models were adjusted by sex, categorical age, race/ethnicity, education, poverty income ratio, dental attendance pattern, and total energy intake.

**Table 3:** Negative binomial regression models for the associations of amount and frequency of added sugars intake with dental caries in US adults (*n* = 10,514).

Outcome	Indicator of Added Sugars Intake	Estimate	Model 1 ^a^	Model 2A ^a^	Model 2B ^a^
DMFS	1-SD change in amount	Rate Ratio	1.12	1.11	1.11
	(54.5 g/day)	[95% CI]	[1.08–1.16]	[1.08–1.15]	[1.07–1.15]
		*p*-value	<0.001	<0.001	<0.001
	1-SD change in frequency	Rate Ratio	1.06	1.02	
	(2.3 items/day)	[95% CI]	[1.03–1.09]	[0.99–1.04]	
		*p*-value	<0.001	0.301	
	1-SD change in frequency	Rate Ratio	1.07		1.05
	(1.1 episodes/day)	[95% CI]	[1.04–1.09]		[1.02–1.08]
		*p*-value	<0.001		0.001
DS	1-SD change in amount	Rate Ratio	1.41	1.49	1.43
	(54.5 g/day)	[95% CI]	[1.32–1.52]	[1.38–1.61]	[1.33–1.54]
		*p*-value	<0.001	<0.001	<0.001
	1-SD change in frequency	Rate Ratio	1.09	0.91	
	(2.3 items/day)	[95% CI]	[0.97–1.22]	[0.81–1.02]	
		*p*-value	0.135	0.101	
	1-SD change in frequency	Rate Ratio	1.01		0.95
		[95% CI]	[0.92–1.11]		[0.86–1.04]
		*p*-value	0.867		0.269

^a^ Model 1 was adjusted for confounders (sex, categorical age, race/ethnicity, education, poverty income ratio, dental attendance pattern, and total energy intake). Models 2A and 2B were additionally adjusted for the indicator of added sugars intake reported in the table.

## Data Availability

Data could be made available upon a reasonable request to the corresponding author.
